# Experiential Neurorehabilitation: A Neurological Therapy Based on the Enactive Paradigm

**DOI:** 10.3389/fpsyg.2020.00924

**Published:** 2020-05-15

**Authors:** David Martínez-Pernía

**Affiliations:** ^1^Center for Social and Cognitive Neuroscience, School of Psychology, Adolfo Ibáñez University, Santiago, Chile; ^2^Geroscience Center for Brain Health and Metabolism (GERO), Santiago, Chile; ^3^Memory and Neuropsychiatric Clinic (CMYN), Neurology Service, Hospital del Salvador and Faculty of Medicine, University of Chile, Santiago, Chile

**Keywords:** enaction, embodied consciousness, experiential neurorehabilitation, cognitive paradigm, cognitive neurorehabilitation

## Abstract

With the arrival of the cognitive paradigm during the latter half of the last century, the theoretical and scientific bases of neurorehabilitation have been linked to the knowledge developed in cognitive neuropsychology and cognitive neuroscience. Although the knowledge generated by these disciplines has made relevant contributions to neurological therapy, their theoretical premises may create limitations in therapeutic processes. The present manuscript has two main objectives: first, to explicitly set forth the theoretical bases of cognitive neurorehabilitation and critically analyze the repercussions that these premises have produced in clinical practice; and second, to propose the enactive paradigm to reinterpret perspectives on people with brain damage and their therapy (assessment and treatment). This analysis will show that (1) neurorehabilitation as a therapy underutilizes body-originated resources that aid in recovery from neurological sequelae (*embrained therapy*); (2) the therapeutic process is based exclusively on subpersonal explanation models (*subpersonal therapy*); and (3), neurorehabilitation does not take subjectivity of each person in their own recovery processes into account (*anti-subjective therapy*). Subsequently, and in order to attenuate or resolve the conception of embrained, subpersonal and anti-subjective therapy, I argue in support of incorporating the enactive paradigm in rehabilitation of neurological damage. It is proposed here under a new term, “experiential neurorehabilitation.” This proposal approaches neurological disease and its sequelae as alterations in dynamic interaction between the body structure and the environment in which the meaning of the experience is also altered. Therefore, when a person is not able to walk, remember the past, communicate a thought, or maintain efficient self-care, their impairments are not only a product of an alteration in a specific cerebral area or within information processing; rather, the sequelae of their condition stem from alterations in the whole living system and its dynamics with the environment. The objective of experiential neurorehabilitation is the recovery of the singular and concrete experience of the person, composed of physical and subjective life attributes.

## Introduction

People who suffer brain injury (stroke, trauma, tumor, neurodegeneration, etc.) may be left with sequelae lasting days, weeks, and years – or their entire lifetime. As the World Health Organization states, through the international classification of functioning, disability and health (ICF) (World Health Organization, 2001), these consequences affect anatomical structure and physiological and psychological functions (Body Functions and Structures), the performance of individual tasks (Activity) and social interaction and development (Participation). The impact of these sequelae is so great that the person may stop taking care of themselves (dressing, eating, walking) and suffer loss of family, work and social environment.

The Health Sciences discipline dealing with recovery from brain injury sequelae is called neurorehabilitation, defined as “a systematic, functionally oriented service of therapeutic activities that is based on assessment and understanding of the patient’s brain-behavioral deficits” (Cicerone et al., 2000, pp. 1956–1957). In recovering from sequelae, neurorehabilitation maintains a multidisciplinary approach where different clinical therapeutic perspectives work toward biopsychosocial recovery. Mainly, disciplines like neuropsychology, physiotherapy, occupational therapy, and speech/language therapy have field-specific actions for behavioral rehabilitation^1^. For example, in neuropsychology, behavior is physical action in performing daily activities, or behavior occurring during a given cognitive task; physiotherapy, meanwhile, sees behavior as physical action in balancing and walking under any condition in which it may occur (automatic, conscious, or in interference with other cognitive tasks); in occupational therapy, behavior is physical action in self-care (grooming, feeding, dressing, moving, toilet training); and for speech and language therapy, behavior is personal ability to appropriately understand and communicate ideas using spoken and written language. Despite differences, the ultimate goal of each discipline is “increasing or improving an individual’s capacity to process and use incoming information so as to allow increased functioning in everyday life” (Sohlberg and Mateer, 1989, p. 3).

For over two millennia, rehabilitation of people with neurological damage was based on the recovery of the physical structures of the body – without consideration for mental processes (Martínez-Pernía et al., 2017). With the arrival of the cognitive paradigm during the latter half of the last century, however, the theoretical and scientific bases of neurorehabilitation have been linked to the knowledge developed in cognitive neuropsychology and cognitive neuroscience (McMillan and Greenwood, 1987; Gianutsos, 1989; Sohlberg and Mateer, 2001; Wilson, 2002; Wilson and Gracey, 2009; Prigatano, 2013). With the scientific premises of these disciplines, rehabilitation methodology was constructed to provide clarity on the nature of cognitive disorders themselves while implementing rehabilitation programs to stimulate specific responses at the brain level to improve behavior and biopsychosocial recovery.

Although the knowledge developed in neurorehabilitation – through the disciplines of cognitive neuropsychology and cognitive neuroscience – has generated very relevant contributions to neurological therapy, this manuscript presents a critical analysis of its theoretical premises. The present manuscript has two main objectives: first, to explicitly set forth the theoretical bases of cognitive neurorehabilitation and critically analyze the repercussions that these premises have produced in clinical practice; and second, to propose the enactive paradigm to reinterpret perspectives on people with brain damage and their therapy (assessment and treatment). The cognitive theory will be shown to have had three central repercussions, termed *embrained therapy*, *subpersonal therapy* and *anti-subjective therapy*, each directly influencing interpretations of therapy and the clinical resources used. Subsequently, and in order to attenuate or resolve the conceptions of the cognitive paradigm in neurological therapy, this paper proposes the enactive paradigm as a new theoretical model applicable to neurorehabilitation. The therapeutic proposal presented here, experiential neurorehabilitation, extends the understanding of therapeutic processes to the whole living system and its dynamics with the environment, where the subjective experience of the person plays a relevant role.

## The Influence of the Cognitive Paradigm in Cognitive Neurorehabilitation

The cognitive paradigm has influenced neurorehabilitation through two main models. The first, developed from the field of cognitive neuropsychology, sees the mind as the software of a computer, processing and manipulating information like a program would. This model, known as the “computational metaphor” (Boden, 1979), looks to understanding how the mind processes information without referring to the physical processes of the brain itself (Coltheart, 2001). Psychologist Ulric Neisser, anticipating the cognitive paradigm, defined the mind as a system that processes information in which “sensory input is transformed, reduced, elaborated, stored, recovered and used” (Neisser, 1967, p. 4) in generating appropriate behavior. The cognitive neuropsychology approach has been useful in neurorehabilitation by identifying cognitive deficit, explaining behavioral problems in terms of information processing, and predicting behaviors based on these problems (Coltheart et al., 1994, 2005; Coltheart, 2002; Wilson and Gracey, 2009). Since the vision of cognitive neuropsychology was considered insufficient, the second of these models introduced a new approach to the mind in neurorehabilitation from the field of cognitive neuroscience. This second perspective seeks to reduce the complexity of neurological lesions by studying them exclusively as alterations in information processing (Wilson, 1997, 2002). This new focus based on the knowledge of brain biology, became the “brain metaphor” model of neurorehabilitation (Rumelhart and MacClelland, 1986), and incorporated cognitive impairment into the study of rehabilitation to explain the selective activity of certain cerebral areas and cerebral cooperation processes in performing behavior (Martínez-Pernía et al., 2017).

Although both models make their respective significant contributions in explaining cognition, this manuscript presents a critical analysis that equally affects both perspectives, and therefore, neurorehabilitation. Three critical analyses of the cognitive premises of neurorehabilitation will be presented in the next three sub-sections. It will also explain how each directly restrict interpretations of sequelae and thus limit therapeutic and scientific approaches. I term these perspectives *embrained therapy*, *subpersonal therapy*, and *anti-subjective therapy*.

## Embrained Therapy

A first limitation of the cognitive paradigm within neurorehabilitation is the slight relevance given the body in cognitive processing. By situating cognition and its disorders as an exclusive property of the brain, such rehabilitation models consider other body structures physical entities with no mental properties. Everyday activities such as walking, grooming, reading the newspaper, chatting with friends, or planning the day’s agenda are cognitive processes that happen exclusively in the head – and here, events outside the brain structure have no relevance to mental processing or therapeutic recovery.

Cognitive neurorehabilitation gives so little relevance to the body in rehabilitating cognitive deficits due to its cognitive assumptions, which restrict mental properties to neuronal events located in the head. Indeed, paraphrasing philosophers like Shaun Gallagher, cognitive theory reduces the body to the reception of environmental stimuli to later be used by cognition or its representation in the somatosensory cortex (Gallagher, 1995, 2005). Hilary Putnam affirms that functionalism, a philosophy supportive of the cognitive paradigm, sees cognition as reductionist, where “the person’s brain (your brain) has been removed from the body and placed in a vat of nutrients which keeps the brain alive” (Putnam, 1981, pp. 5–6). In the words of Lawrence Shapiro, cognition is “*envatted*” (Shapiro, 2004, p. 169). Giovanna Colombetti furthermore characterizes the cognitive paradigm as brain-centrism, that is, a model where cognition is situated in the processes that happen in the brain (Colombetti, 2014). In terms very similar to these, neuroscientist Antonio Damasio affirms that, in this theoretical proposal, the mind is embodied but only in cerebral terms. To express this idea he coined the term “*embrained mind*” (Damasio, 1994, p. 118).

Although many authors across various fields of knowledge lament the lack of attention given the body following the emergence of cognition, rarely has it been discussed, nor indeed analyzed, for its impact on clinical application (Martínez-Pernía et al., 2016). Since many interventions require the stimulation of the body for the stimulation of cognitive function, perhaps the assertion that the body has little relevance in cognition and, therefore, in behavior, may be seen with skepticism among neurorehabilitation researchers and therapists. However, since the cognitive paradigm sees the brain as the only relevant biological substrate to be rehabilitated and diagnosed in the person with cognitive impairment, it implies that other biological structures are second-class elements. As such, the principles that undergird recovery processes in cognition and associated functionality (walking, feeding, communication, decision making, etc.) show that corporeality is little, or not at all, taken into account.

Based on the theoretical background of the cognitive paradigm, the only relevant biological substratum that needs to be rehabilitated and diagnosed in a person with cognitive impairment is located in the brain. Here cognitive neurorehabilitation has been effective in the recovery of cognitive and functional deficits, to the extent that it does rehabilitate physical and mental events that occur within the brain, as long as the role of the body is marginal. Under this therapeutic paradigm, the body and the environment are reduced to a set of sensory stimuli that send information to the brain and are simple pathways for the execution of behavior and body signals are not part of the cognitive processing included in deficit recovery – they are merely physical or chemical activities which lack any type of mental property. Regardless of whether the therapeutic intervention is performed in a hospital room or with the presence of loved ones, or whether body stimulation is performed in exteroceptive or proprioceptive sensory systems, this paradigm restricts all body and environmental information to sensory inputs that travel throughout our biology without possessing any cognitive property.

Briefly, and through the above perspective, the stages of intervention for behavioral change have been designed as follows: (1) environmental and bodily stimuli are transported to the brain through the subsequent afferent sensory pathways (bottom–up information); (2) this information, purely physical, changes its properties upon reaching the brain to a functional brain state which simultaneously possesses physical and mental properties – here, in the brain, and not before, cognitive processing and cerebral activity necessary for the rehabilitation of the person takes place-; and (3), once the processing stage is finished, the cognitive information is again reduced to purely physical, corporeal components through the efferent motor pathways (top–down information), giving rise to the expected behavior in the subject.

This argument gives the body little importance as a therapeutic tool in the recovery of cognitive lesions. A paradigmatic example of this disinterest can be found in the disciplines of speech/language therapy and neuropsychology, in which therapeutic intervention consists of modifying information or neuronal processing produced in the brain^2^. To wit: the gold standard of these disciplines is to perform therapeutic sessions with the patient seated in a chair (Martínez-Pernía et al., 2016).

This is not to suggest that bodily or environmental stimulation produced by the therapist during the session – and transferred to the brain as a sensory signal – does not improve neurological damage suffered by the person. Rather, the crux of the discussion is that neurorehabilitation considers that improvements of cognitive deficits are exclusively produced in the cognitive processing at the cerebral level, obviating corporeal and environmental attributes. While neurorehabilitation disciplines apply a wide range of physical and environmental stimuli in order to improve deficits caused by brain injury, the brain representation of these stimuli is reduced to the somatosensory cortex (brain homunculus); nowhere in cognitive explanations thereof is any detail on how environmental and body information have a physical/cognitive brain representation, or what type of cognitive processing is involved.^3^

As seeing appropriate behavior produced through storing, filtering, encoding and retrieving information, this intervention model still lacks an explanation of what specific characteristics of the environment and the body are incorporated into cognitive and brain processing. Even in ecological therapeutic intervention approaches, where the person performs physical actions in a given environment (shopping in a supermarket, paying bills in a bank branch, chatting with several people while walking through the park, climbing the escalators of a shopping mall, etc.), the marginality of corporeality as a whole is still present.

The explanations offered by cognitive neuropsychology and cognitive neuroscience – modular systems of cognitive processing implemented in brain neurobiology-, and therefore neurorehabilitation practices based thereupon, are insufficient. It reduces the explanation of clinical improvement to the recovery of cognitive and brain structures, and relegates the body and the environment to non-mental sensory physical events. Borrowing Damasio’s neologism, neurorehabilitation is a therapy embrained.

In sum, the theory under which neurorehabilitation is governed not only has implications in the way cognition is explained in the rehabilitation of deficits; rather, its assumptions further generate pre-theoretical determinants in the way therapy implements clinical intervention. Any therapy based on the scarce relevance of the body in cognition will be doomed to generate research models or clinical interventions in which the attributes of the body are not taken into consideration or in which it is given scarce relevance.

## Subpersonal Therapy

A great advance of the cognitive paradigm against previous mechanistic assumptions was that of opening the “black box.” Although behaviorism gave a satisfactory explanation of the learning of new behaviors, it was never able to account for the underlying mental processes. The cognitive paradigm, in turn, gave access to the internal processes preceding behavior, which, for cognitive neurorehabilitation, occur in terms of information processing or brain activity. In spite of such advances, some authors have commented that the approach has generated new problems in mind research because it produces a stagnation in certain behavioral precepts. John Searle put it this way: “Cognitive science promised a break with the behaviorist tradition in psychology because it claimed to enter the black box of the mind and examine its inner workings. But unfortunately most mainstream cognitive scientists simply repeated the worst mistake of the behaviorists: they insisted on studying only objectively observable phenomena, thus ignoring the essential features of the mind. Therefore, when they opened up the big black box, they found only a lot of little black boxes inside” (Searle, 1992, p. xii).

The “little black boxes” referred to by Searle are the representation of the mind in terms of events inaccessible to conscious experience. Although the cognitive paradigm managed to explain what happens in the mind between the presentation of the stimulus and the production of the behavior, its weakness lies in the fact that its explanation takes place in terms of processes that are unapproachable by the consciousness. This perspective explains only what type of processing is required by the information that enters the system, and the neurobiological activity that takes place within it, without requiring individual consciousness information as experienced and expressed.

Succinctly, the cognitive paradigm considers the mind a non-conscious process, hidden to the singular and cognizant perspective of the individual (Dennett, 1969; Sacks, 1985; Bruner, 1990; Jopling, 1996). The study of cognition and its understanding depends on information processing, neurobiological activity, electrical activity, serial information processing, and cerebral blood flow: processes all unintelligible to, and inaccessible to, the conscious experience of the individual. In other words, “what makes experience possible in the first place is not itself a possible object of direct experience” (Jopling, 1996, p. 158). Dennett (1969) stated that the cognitive paradigm maintains a *subpersonal* explanation, where personal explanations have no relevance, coining the term. That is, explanation is dependent on patterns of brain activation or functional organization with no room for the subject’s singular perspective (belief, desire, thought).

The repercussions of this mental model based on subpersonal explanations go beyond low-level cognitive processes (attention, memory, perception, comprehension of language, etc.). The cognitive paradigm also explains high-level cognitive processes (thinking, reflection, decision making, awareness, executive function, metacognition) with its model of the unconscious mind (Reber, 1992). The elements of cognition required for analysis, interpretation, observation, evaluation or judgment still occur outside of a subject’s conscious experience. It is only the end result of cognitive processing a person has access to, not the cognitive process itself. Miller, expressed that consciousness “is the result of thinking, not the process of thinking, that appears spontaneously in consciousness” (Miller, 1962, p. 56); Mandler, that “the analysis of situations and appraisal of the environment goes on mainly at the non-conscious level. There are many systems that cannot be brought into consciousness, and probably most systems that analyze the environment in the first place have that characteristic. In most of these cases, only the products of cognitive and mental activities are available to consciousness.” (Mandler, 1975, p. 245); Neisser expressed that constructive processes “themselves never appear in consciousness, their products do” (Neisser, 1967, p. 301); and Alexander Luria, father of modern neuropsychology – and who believed the study of the mind was being reduced to questionnaires, mathematical schemes, and devices that measured brain activity (Jopling, 1996; Good, 2000) – stated that “the reality of human conscious activity was being replaced by mechanical models” (Luria, 1979, p. 176).

Transferring this analysis to the discussion of how subpersonal models have influenced neurorehabilitation, these precepts for understanding, investigating, and exploring mental phenomena and conscious experience reduce rehabilitation intervention models to focus purely on cognitive processing and brain activity via determinants of automatic cognitive processes and the ability to self-referentially manipulate symbols. In showing that cognitive neurorehabilitation assumes the ontology of the mind as a subpersonal process, let us discuss this precept’s influence on neurorehabilitation, both research and therapy, below. To do so, I distinguish two models of subpersonal therapy neurorehabilitation. The first is “subpersonal therapy *sensu stricto*”; and the second, “subpersonal therapy *sensu lato.*”

### Subpersonal Therapy Sensu Stricto

Psychologist and Nobel Prize in Economics Kahneman (2011) described thinking as system 1 and system 2. System 1 includes all mental processes that operate quickly, automatically, stereotypically, unconsciously and implicitly; and system 2, slow, infrequent cognitive processes that require effort on the part of the subject, are conscious, and have a logical or calculating character. In terms of cognitive domain, system 1 is attributed low level cognitive functions such as attention, perception, comprehension of language, memory or visual construction, among others. On the other hand, system 2 is related to high-level cognitive processes such as sequencing, planning, decision-making, reflection, thinking, working memory and impulse control, among others.

Taking Kahneman’s analysis to a personal context, low-level information processing is so fast that the person spontaneously produces the output of the task “automatically.” For example, when a person is shown the image of a lion and asked what that animal is called, suddenly, and without being able to explain how, they find in their head the mental content “Lion.” On the other hand, perception of high-level cognitive processing varies substantially, in which it is assumed that the person is capable of handling and manipulating symbols once all unconscious processing has finished. That is, a person symbolically handles mental representations and manipulates them, it bears repeating, via subpersonal cognitive processing. A clinical example is asking patients the sum of 11, 17, and 24. In order to obtain the answer to this question, the patient must manipulate the different mental contents that appear in their head in order to be able to carry out the task effectively.

Whereas the two systems described by Daniel Kahneman differ substantially, both in terms of neurobiology and patient perception, therapeutic intervention models developed therefrom take similar approaches. Neurorehabilitation under a subpersonal framework ignores personal and individual positions on how to solve cognitive tasks. These therapeutic interventions cannot access the possibility of implementing strategies related to individuality, and exclude meaningful learning, divergent thinking, creativity, emotionality or exploration of new behaviors. The perspective assumes that the person (i.e., in this case, the brain) correctly performs the cognitive task through natural qualities necessary to solve the problem. During clinical intervention, the subject is not expected to learn through certain individual dispositions that may allow them to face the task from their own position. Rather, the subpersonal laws of neurobiology expect the patient to resolve the proposed cognitive task in which the qualities of the physical world are innately present, with no sign of subjectivity. An example of the therapeutic strategies governed under this subpersonal model is *errorless learning*, tirelessly repeating the same semantic or phonetic tracks in order to automate learning, stimulate senses, and generate habits and routines.

### Subpersonal Therapy *Sensu Lato*

While it is true, as shown above, that the cognitive paradigm does not take personal perspectives into consideration, there is yet a paradox: a multitude of research paradigms (cognitive tasks) and clinical interventions (assessment and treatment) require participant self-awareness^4^. Under this high-level cognitive process, the participant self-explores their own mental contents through introspection. This is subpersonal therapy *sensu lato*, where the therapeutic effect appears once the person appropriates their mental contents and discovers an element of which he or she was previously unaware.

Although cognitive neurorehabilitation, from its subpersonal proposal, affirms that self-consciousness is the final product of cognition and that, therefore, “it could not be a cause of anything” (Bruner, 1990, p. 9), under this therapeutic methodology it is affirmed at the same time, although implicitly, that the personal dimension is a relevant factor implied in the person’s recovery mechanism. It is in the very act of “awareness” where the explanation of cognition as a subpersonal process is insufficient and where the subjective and personal discovery of a new element in the consciousness and for the consciousness appears as a necessary mechanism of the recovery process.

Unlike other high-level cognitive processes in the previous section, it is in this intervention based on awareness that is found in subpersonal therapy *sensu lato*. From this methodology the therapy can no longer be explained in terms of subpersonal processes and needs the very act of self-awareness to make the therapeutic change understandable. In other words, the process of characterizing the subjective cannot be reduced to any other level of explanation. Examples of this type of intervention include feedbacks (verbal, visual, audiovisual), drawing a performance graph, declarative presentations of personal deficits, writing strengths and weaknesses of performed tasks, real world experiences, positive reinforcement, the use of non-confrontational discussions (patient and therapist) about the performance of the task, and self-evaluation systems (Lucas and Fleming, 2005; Cheng and Man, 2006; Fleming and Ownsworth, 2006; Ownsworth et al., 2006; Toglia et al., 2011; Schrijnemaekers et al., 2014).

While the conceptual understanding of subpersonal therapy *sensu lato* allows for a better understanding of the characteristics of awareness-based therapy, its intervention model still carries certain cognitive premises that limit therapeutic processes. Below, I briefly explain three types of awareness therapy reductionisms from traditional cognitive neurorehabilitation methodologies.

#### Mental Content

Therapeutic interventions meant to modify mental content in participants focus efforts on the individual discovering their mental content in order to acquire new content endowed with characteristics that minimize sequelae. This cognitive paradigm, however, reduces consciousness to a construct found in the mental image, and does not explore the possibility that consciousness may be constituted of processes present before the elaboration of the mental content or underlying it.

#### Introspection

This neurorehabilitation methodology looks to access mental content through introspective acts; that is, the subject must turn their mind toward themselves (re-flection, or turning into oneself) to understand their mental content, which reduces alternatives for exploring one’s own experience. Therapeutic success is measured by the exercise applied during therapeutic sessions (speaking, evaluating, comparing, drawing performance), and does not include the processes themselves involved in accessing or creating mental content.

#### Rationality

Neurorehabilitation assumes that recovery is subsumed to a rational mind accessing its mental contents. Patient ability for awareness is placed under the domains of rationality and, as such, strategies work to help the patient logically understand the mental events to which they do not have access (thinking, believing, reflecting, arguing, evaluating, comparing). This proposal is reductionist by not including a pre-reflective look at the constitution of consciousness, i.e., at the point where personal knowledge is created by events prior to the conformation of their rational world.

Based on the rational mind, introspection, and access to mental content, it is unquestionable that this clinical intervention model has contributed significantly to neurorehabilitation. Notwithstanding, it remains reductionist in the understanding of consciousness. It limits the rich spectra of mental attributes and the potential diversity of clinical therapy interventions.

## Anti-Subjective Therapy

The third ontological repercussion presented in this work refers to the restriction of attributes with which the mind is defined or characterized. In order to delve deeper into this idea, I will now explain how neurorehabilitation understands subjectivity in a person with neurological lesions, and what repercussions such premises have on therapeutic intervention.

One of the great philosophical criticisms against the cognitive paradigm is centered on its definition of the essence of what is human (e.g., Gallagher, 2005; Gallagher and Zahavi, 2008; Colombetti, 2013). Even at its earliest, this paradigm was criticized by many for its concept of mind, here, lacking attributes that relate cognition to the existence of a unique and singular individual. The development of the theoretical and scientific program of cognitivism abandoned subjective attributes for information processing and brain activity. This model of cognition redefines the qualities that make us “human,” and has been harshly criticized by philosophers and neuroscientists for legitimizing subpersonal processes without reference to personality, identity, consciousness, emotion, belief, desire, volition, motivation, or meaning. The philosopher David Jopling explained it in the following way: “The postulated entities and systems forming the explanans of sub-personal theories bear none of the familiar identifying marks of consciousness, selfhood or personality: the systems are anonymous, impersonal and thin” (Jopling, 1996, p. 159); and Matthew Elton argued that “Consciousness is a product of certain capacities that are intelligible only at the personal level, capacities that are neither present at the sub-personal level of brain mechanism nor present in ‘sub-persons”’ (Elton, 2000).

In addition to these criticisms from the basic sciences, different therapy professionals have harshly criticized the anti-subjective cognitive model. Thus, for example, the post-rationalist psychologist Juan Balbi explained it in the following terms: “The computational conception of the mind does not contemplate its subjective and intentional character and excludes the possibility of a scientific explanation of human consciousness and self-awareness. By adopting as a computational ‘metaphor of mind’ model, cognitive psychology has turned toward a new kind of anti-mentalism, more subtle and technologically equipped and, perhaps, even more vigorous than the previous [behaviorism]” (Balbi, 2004, p. 184). Another important and remarkable figure in cognitive psychology who strongly criticized the anti-subjectivist vision was Alexander Luria. Although Luria was one of the most influential psychologists in the theory of cerebral organization and behavior with his works on aphasia (Luria, 1970) and higher cognitive functions (Luria, 1966), his particular therapeutic vision saw an important limitation in the cognitive proposal vis-à-vis its abandonment of the study of subjectivity. To quote a letter from Alexander Luria addressing the eminent neurologist Oliver Sacks, in which he clearly criticizes the anti-subjective therapy model: “There are no prescriptions in a case like this. Do whatever your ingenuity and your heart suggest. There is little or no hope of recovery in his memory. He has feeling, will, sensibilities, moral being. Matters of which neuropsychology cannot speak. And it is here, beyond the realm of an impersonal psychology, that you may find ways to touch him, and change him. Neuropsychologically, there is little or nothing you can do, but in the realm of the Individual, there may be much you can do” (Sacks, 1985, p. 32).

In the field of neurorehabilitation, anti-subjectivity has impacted therapeutic methodology and the concept of the patient during the therapeutic intervention. To this end, let us explore what the denial of the attributes of subjectivity consists of in the person who turns to neurorehabilitation (“*the anti-subjective person*”); and, then, with what type of attributes neurorehabilitation replaces subjectivity in its participants (“*the impersonal*”).

### The Anti-subjective Person

As in subpersonal therapy, the cognitive paradigm only considers therapeutic strategies as useful when they act at the level of unconscious processing. Patient recovery is based on strategies focused on neuronal stimulation, brain plasticity, the generation of new information processing routes, or the recovery of information that, until now, had not been available. They make use of tools based on a methodology that omits any reference to the personal characteristics of the patient, such as the will, eagerness to overcome, responsibility, anger, hope, spirituality, faith, motivation, morality, etc. All of these are unique and singular attributes of each human being which may facilitate or impede quality of life, biopsychosocial recovery, and success in the rehabilitation program.

All therapeutic disciplines of cognitive neurorehabilitation (physiotherapy, occupational therapy, speech therapy, psychology) apply interventions whose purpose is to restructure the cognitive and cerebral system, regardless an individual’s personal history, the experiences that shape their present, or elements that will make their look at the future hopeful or heartbreaking; intervention is reduced to influencing unconscious subpersonal processes, where the person with brain damage is subordinate to therapy. Under these anti-subjective premises, cognitive neurorehabilitation considers that the learning process is produced by means of constant and repetitive stimulation of cognitive processes in the consolidation of which the subjective experiences of the patient have no repercussion whatsoever. Neurorehabilitation overlooks in its theory of learning those elements that for the patient are deeper or more full of personal meaning, learning loaded with a subjective quality that could facilitate the process of therapeutic recovery.

This model of intervention may have dramatic consequences. An anti-subjective approach dismisses suffering, will, personal improvement, dignity in the face of illness, or the shame of feeling ill. Neurorehabilitation abandons central aspects of our existence, making it a therapeutic model far removed from our humanity. This devaluation of the subjective dimension undermines clinical practice and reduces therapeutic intervention to subpersonal process determinants. Even strategies based on self-awareness, which implicitly assume the existence of a personal level, suffer from this subjective dimension. From the perspective of an awareness task, subject access to their mental content is recorded as a binary (yes/no), disregarding any personal accompaniment to the experience (frustration, anger, happiness, neglect).

### The Impersonal

Impersonal therapy, instead of showing the individual as an entity full of “internal,” personal experiences, describes them as a set of cognitive processes sans subjective qualities. Whatever the personal explanation presented, it is never in terms of individual attributes or personal qualities, but rather of how the subject is able to organize and elaborate information and behavior – an explanation based on the *impersonality* of the person with brain damage. Under this therapeutic approach, the person ceases to be a subjective entity and becomes an impersonal entity. It is an explanation that does not need any reference to individuality, where everything is expressed in terms that disregard subjective life attributes of the person.

By causing the person with brain injury to be seen not as a sentient entity loaded with attributes that make them unique, but as a logical entity that processes information in an efficient and objective manner – a function of computational algorithms in cerebral/subpersonal systems – the cognitive interpretation of the human being is logical, rational, and objective. Cognitive neurorehabilitation has incorporated this as its impersonal therapeutic model, where there is only room for attributes constructed under scientific rationality, reducing cognition to attention, memory, perception, language, visual construction, praxis, locomotion, and executive function^5^. This approach of neurorehabilitation has developed a corpus of knowledge that denies subjective attributes and replaces them with a rational and objective vision of the therapeutic process and cognition: this is exemplified in neurorehabilitation diagnoses.

Indeed, diagnosing these domains implies accepting an intellectualist vision of the patient’s world, a vision of reality and therapy mediated by rules, norms, and laws that avoid any reference to the interiority of the person (self, consciousness, self-awareness, volition, motivation, emotion, meaning). This therapeutic discipline perspective maintains an intellectualistic vision of the world, where people must adopt an impersonal attitude regarding the task assigned to them. For example, the verbal fluency “P” test requires enunciating a minimum of words beginning with “P.” A person successful in the task is able to shift between different strategies in searching for words. Another example of assessment is the clock test, where a person is asked to draw a clock with their face and 12 numbers in their correct position. Processing this cognitive-motor task correctly suggests their visual perceptive ability is unharmed.

Cognitive assessment is based on the fact that the patient must approach the cognitive task under the premises of objectivity, planning, sequencing, reflection and evaluation, all of which are characterized by the absence of the subjective quality of the participant within the task posed, and replaced by an impersonal vision of the world that surrounds them.

## The Enactive Paradigm

In the previous section, the repercussions of the cognitive paradigm on neurorehabilitation were presented. First, it was argued how neurological therapy underutilizes interventions focused on corporeality and an explanatory model that focuses exclusively on cognitive and cerebral processing. Next, it was shown how neurorehabilitation assumes as possible variables of the therapeutic and research process those that are inaccessible to the consciousness of the patient and the therapist or researcher. Finally, and directly related to a subpersonal explanation model, it was argued that neurorehabilitation lacks a therapeutic model in which there is room for a therapy based on subjectivity. Given these three repercussions, which in the light of this manuscript restrict the assessment, therapeutic, investigative and recovery process of the person with brain damage, this section will present the enactive paradigm as an ontological proposal that could minimize or overcome the limitations previously mentioned.

Currently, enaction is considered a new paradigm in cognitive sciences (Stewart et al., 2010) constituted by different approaches. Following the categorization of phenomenologist Shaun Gallagher these approaches are called “the 4e approaches of the mind” and where cognition is considered to be Embodied, Enacted, Embedded, and Extended (Rowlands, 2010). The enactive paradigm has been widely applied in various fields of knowledge such as neuroscience, philosophy, education, psychology and artificial intelligence, among others (Damasio et al., 1991; Varela et al., 1993; Brooks, 2003; Gallagher, 2005; Shapiro, 2011; McGann et al., 2013). However, in the field of neurorehabilitation few studies have been developed from this perspective (Martínez-Pernía and Ceric, 2011; Øberg et al., 2015; Hay et al., 2016; Martínez-Pernía et al., 2016; Repetto et al., 2016; Cardona, 2017).

The theoretical position that defends the enactive paradigm, as opposed to the cognitive paradigm, is the denial that the mind can be explained from a materialistic reductionism that limits any explanatory construct to the physical mechanisms and/or cognitive processes that are located in the head. From the enactive perspective, the body ceases to be understood as a secondary process of the mind. The body is not limited to being a mere physical entity that sends and transmits information from the world to the brain. The enactive proposal converts the body into the necessary substratum from which consciousness emerges and from where attention, memory, reasoning, consciousness, emotion, subjectivity, etc., take shape (Gallagher, 2005). It is a perspective from which the “states of the body modify states of the mind” (Wilson and Golonka, 2013, p. 1). This is how the dynamic interactions between the physiology of the organism, the sensorimotor schemes and the environment allow the development of life and cognition (Varela et al., 1993; Thompson and Varela, 2001). In opposition to the cognitive paradigm, which prioritizes the brain over any other biological dimension, the enactive paradigm affirms that the body, the environment and the brain are constituted by a structural coupling that cannot be divided or sectioned in its study and in which all of them have equally shared responsibility for the emergence of the mind (McGann et al., 2013). Therefore, the living organism, mind and environment are indissolubly intertwined properties in cognition that require simultaneous research (Thompson, 2007). The enactive paradigm, and therefore a neurorehabilitation based on that paradigm, proposes to abandon the concept of the body as an empty substance and identify it with an existential biology, a biology with meaning and personal sense. From this perspective, the concept of cognition as a subpersonal process disappears and is replaced by a model of consciousness based on the philosophical current of phenomenology (Varela et al., 1993; Gallagher, 2005; Thompson, 2007; Colombetti and Thompson, 2008; Gallagher and Zahavi, 2008; Rowlands, 2010) where bodily correlates are in turn subjective correlates.

## Experiential Neurorehabilitation: a Therapeutic Proposal Based on the Enactive Approach

The purpose of this section is to move the discussion from the enactive paradigm to the field of neurorehabilitation. I show the clinical implications for addressing neurological disorders from enaction, and how therapy is transformed under its premises.

An element to which I would first like to draw attention is that this approach – which has been given different names in the basic sciences^6^, is here termed “embodied consciousness” in its concrete application to the therapeutic sciences. The conceptual precision on which neurological therapy is based is not minor and, in itself, is a declaration of intent. Currently, research carried out under the enactive paradigm has predominantly been from the fields of the basic sciences (Gallagher, 2005). In those disciplines, while research has mainly focused on demonstrating the entanglement among brain, body, and environment, there has yet to be a genuine effort to study the new characteristics of the mental associated with this paradigm (Gallagher and Zahavi, 2008). This oversight is not irrelevant: the neurological therapy based on the embodied consciousness approach requires understanding how a person is conscious of their experience. Unlike the basic sciences, where the main variable for studying cognition is biological, the therapeutic sciences necessarily call studying conscious experience where the biological response (third-person view) and the subjective experience (first-person view) are fundamental elements in rehabilitating a person with neurological damage. Currently, different authors of the enactive paradigm emphatically and explicitly defend that the study of the mind in this paradigm is based on the precepts of phenomenology^7^ (e.g., Varela et al., 1993; Gallagher, 2005; Thompson, 2007; Colombetti and Thompson, 2008; Gallagher and Zahavi, 2008; Colombetti, 2014).

In order to make use of a vocabulary that distinguishes this therapeutic model from others, I propose the term “experiential neurorehabilitation” to designate neurological therapy based on the embodied consciousness approach. Continuing with conceptual clarifications, “experiential,” as applied here, is far removed from the panpsychist proposals of some therapeutic approaches (e.g., humanistic-existential therapy). Rather, in the context of a therapy based on embodied consciousness, the term “experiential” refers to the constitution of a human being who is in the world – natural and social – with corporeal and intentional attributes.

If I transfer the embodied consciousness approach to the very definition of neurological injury and the consequences it has in the life of the person (body functions and structures, activity, and participation) important contributions are observed under the proposal of embodied consciousness. Under this perspective, the neurological disorder and its consequences are not only alterations in the processing of information or deficits in the patterns of brain activity that underlie the behavior. In addition, this proposal states that brain damage and its consequences are a disorder that is situated in the process of dynamic interaction between the body structure and the environment that surrounds it. And where the subjectivity of the agent is part of this dynamic. Therefore, when a person is not able to walk, communicate or maintain an efficient self-care, their deficits are not only a product of the alteration of a certain cerebral area or the functional state of the brain. The sequelae of his condition are caused by the alteration of the dynamics of the body–environment and in which the meaning of the experience is also altered.

To illustrate the importance of an paradigmatic shift in the neurorehabilitation from cognitive to embodied consciousness perspective, let us see how Parkinson’s disease is defined according to each, using as a case in point the following visual recording of a person with advanced stage Parkinson’s disease (Snijders and Bloem, 2010). The first part of the video shows a traditional gait assessment setting, where the person has enormous difficulties in walking a few meters along a hospital corridor. From the cognitive paradigm, this symptomatology is a product of the death of dopaminergic nervous cells in the *pars compacta substantia nigra* (Kalia et al., 2015). The second part of the video shows a totally different phenomenon related to Parkinson’s symptoms – the same person, this time pedaling a bicycle down the street, turns to return to the point from which they left, and is even able to pedal standing on the bicycle without any support from the saddle. The cognitive paradigm explains away this phenomenon by stating that the person has retained motor schema related to that part of the physical activity intact. Experiential neurorehabilitation, on the other hand, offers a more versatile perspective regarding variations of neurological sequelae through its contextual and changing approach to disease. Under this perspective, patient symptomatology is a dynamic process that changes according to the body’s interaction with the environment and its subjective experience, in such a way that said neurological sequelae are expressed differently between walking down a hospital corridor and pedaling a bicycle up the street. Experiential neurorehabilitation conceives of neurological pathologies and consequences not only as exclusively individual-cerebral disorders, but depending upon the temporal immediacy of brain, body, environmental, and subjectivity dynamics.

Another alternative in implementing experiential diagnosis considers corporeality as a dynamic of sensorimotor interactions with others; that is, neurological disease is explained by analyzing body dynamics of intersubjective interaction, and not just “in the head.” Thus, for example, Hanne De Jaegher describes the pathology of autism as the relationship that a person maintains with their social environment from their embodied experience associated with their particular environment (De Jaegher, 2013); while McGann et al. (2013, p. 206) affirm that “a person with autism often functions better in some types of situations than in others. It may be just as plausible to characterize the person-environment situations as problematic, describing the engagement or the interaction as ‘disordered’, and not just the individual.”

In order to deepen the therapeutic perspective from the enactive paradigm, the next section will explain a possible way to interpret experiential neurorehabilitation. For this, I rely on the proposal developed by Shaun Gallagher on the structure of body experience (Gallagher, 1995, 2000, 2005; Gallagher and Zahavi, 2008).

## Therapeutic Principles Based on the Structure of Body Experience

Experiential neurorehabilitation, in line with its theoretical premises, considers the co-existence of two entities that must be taken into account simultaneously during clinical assessment and therapeutic intervention. These two entities are structured into the “prenoetic structure” and the “intentional project” (Gallagher, 1995, 2000, 2005; Gallagher and Zahavi, 2008).

## Prenoetic Structure

Those aspects of consciousness that have no intentional content and are inaccessible to conscious experience are prenoetic structures. These emphasize the importance of the interaction between the environment and corporeality for the formation of consciousness and cognition. This concept understands corporeality as situated in the world prior to perception and action, without reducing to its mere biological dimension. Under such premises, neurorehabilitation abandons the concept of the body as an empty substance, and rather identifies it with a body that contains the meaning of experience without the need for any conscious symbolization. It is a body that carries in itself the quality of experience via immediacy. With the experiential approach, biology carries meaning in each person: only through the body does the meaning of experience appear, integrated into our existence without any reflective process. Bodily existence is a life full of meaning present, before thought, reflection, or self-awareness.

These prenoetic structures function to achieve an adequate coupling process between the environment and the person, automatically, without the need for conscious processes. Applying prenoetic structure constructs in clinical terms has relevant therapeutic implications. Unlike a cognitive model of rehabilitation, where only productions at the level of brain stimulation are relevant, experiential neurorehabilitation emphasizes a scope of intervention into body–environment interaction processes. In other words, while cognitive neurorehabilitation develops a model of therapy where brain stimulation prevails (embrained therapy), experiential neurorehabilitation opens new interpretative paths in therapy by considering the whole body dimension and the environment that surrounds it as relevant, in and of themselves, in recovering from sequelae. Enactive principles have been used in therapeutic intervention previously: for example, gait in Parkinsonian patients’ has been rehabilitated with musical therapy (musical beat, metronome). Indeed, Schiavio and Altenmüller (2015) indicate that locomotory rehabilitation in a musical environment activates concrete sensorimotor dynamics, expressed through bodily interaction with the musical environment that embeds and creates new world of meanings for a person. This form of neurological therapy expands upon reductionist explanations of cognitivism, which sees recovery in walking as a product of cortical sensorimotor network plasticity (Rojo et al., 2011).

By incorporating a new theoretical framework in neurorehabilitation, not only will the understandings of the therapeutic process be broadened to living systems and their environmental interactions as a whole, so too will the possibility of generating and creating new therapeutic strategies based on its theoretical precepts. Thus, for example, the study developed by Martínez-Pernía et al. (2016) investigated differences in behavioral and cognitive performance under two different postural settings (sitting on a chair vs. sitting on a ball). That exploration of patient dynamics in structural coupling between the body and the environment in rehabilitative learning showed that neuropsychological therapy sitting on a ball achieves better cognitive performance and greater behavioral self-regulation than sitting on a chair (traditional therapeutic setting). Performing cognitive tasks on a ball was shown to increase automatic body-balancing resources (prenoetic structure), aid patient focus on the task (intentional project), and reduce attention on irrelevant environmental stimuli.

## Intentional Project

This approach assumes that, at the personal level, the Subject is immersed in a universe of meanings. People continually live experiences of personal and cultural meanings, and indeed experience the world, full of such meanings, with the ability to reflect on one’s own experience. Unlike cognitive neurorehabilitation where experience is reduced to subpersonal and anti-subjective levels, the intentional project holds space for knowledge of a dimension of pre-reflective aspects of experience, as well as of the constitution of the very structures of consciousness. Through the concept of intentionality, all experience is susceptible to self-inquiry and self-exploration, in personal terms. This allows one to go beyond attributes of subpersonal therapy, both *sensu stricto* and *sensu lato*. While low and high level cognitive processes are, in cognitive neurorehabilitation, attributes of the mind under subpersonal processing, experiential neurorehabilitation refers this concept of mind to the unique and individual vision of each person with neurological impairment.

This phenomenological vision the enriches the analysis and study of consciousness, and, unlike subpersonal therapy *sensu lato*, experiential neurorehabilitation holds that the mind cannot be equated to a vision based on the ability of reflexive self-inquiry of its mental contents; rather, this concept entails an unveiling of how mental contents are constituted from their prenoetic and pre-reflective bases. From this perspective, experience is made up of physical, perceptual, temporal, spatial, emotional and meaningful attributes, all of which produce a unique and specific view of ourselves. These characteristics of the mind go beyond the reflexive reductionism (introspection) inherent to subpersonal therapy *sensu lato*.

The phenomenological perspective in experiential neurorehabilitation allows for innovation in the types of strategies applied to clinical intervention, especially those that previously would have made no sense under a framework of cognitive neurorehabilitation. An understanding of the phenomenological mind within the framework of embodied consciousness introduces narration and description of experience as a therapeutic and assessment strategy in phenomenological terms^8^. A subject may access their pre-reflective experiences not only as a consequence of observations of mental content, but also through the self-awareness that appears as the participant contacts their deeper constitutive reality. A person may narrate their experiences, and that narrative becomes part of a self-discovery that does not appear merely through the observation of mental content, but rather emerges from a deeper exercise of internal recollection and “intimacy”^9^ with the object of knowledge.

In the context of these attributes that, at least from the approach of embodied consciousness, constitute experience (i.e., the prenoetic structure and the intentional project) – I may continue to address the discussion that these two attributes should not be understood as isolated entities that shape certain aspects of the experience. That perspective would lead to a new proposal of mind–body dualism. Rather, the prenoetic structure and the intentional project are co-constituent elements of each experience, modified according to their respective characteristics. Experience is not a univocal process of determination, in which prenoetic structures would determine an intentional or conscious project of a subject or the intentional project would determine how the prenoetic structure will carry out its functions; instead, both levels of experience co-regulate themselves to form an experience integrated into a dynamic of structural coupling among environment, embodiment, and underlying subjectivity. These two entities are inseparable to the point that whatever happens in one will also affect the other. For example, although the prenoetic structure functions to achieve an automatic coupling between the environment and the person, its responses are also delimited by the dynamics that occur from the person’s intentional project. In other words, subintentional structures are subject to their own exchanges between the body and the environment and, in turn, conditioned to function through the subject’s intentional project, including possible conscious experiences immediate or proximate to the experience underway. Transferring this theoretical vision to experiential neurorehabilitation will necessarily integrate the whole living system and its dynamics with the environment into therapy intervention models. This therapy would include the prenoetic structure and intentional project as basic elements of neurorehabilitation (assessment and treatment). Take, for instance, the virtual reality episodic memory rehabilitation as proposed by Repetto et al. (2016): sensorimotor interactions of the elderly are increased in a virtual environment (prenoetic structure), where the user has the subjective sensation of being “in action” (intentional project), of experiencing the world from their spatial-temporal experiences (sounds, sensations, perceptions, movements, feelings). Those corporeal experiences, though mediated virtually, will later be central in memory recall (Wilson, 2002).

## Conclusion: the Goals of Neurorehabilitation

The first part of the manuscript presented the cognitive assumptions on which cognitive neurorehabilitation is based as applies to performing neurological therapy, as well as analyses of the consequences that such theoretical assumptions have for interpretations of neurological disease, its sequelae, and therapeutic limitations thereof. Analysis showed that cognitive neurorehabilitation is currently an embrained, subpersonal, anti-subjective therapy. The second part of the manuscript discussed the enactive paradigm and its embodied consciousness approach as an alternative proposal to overcome the limitations of cognitive neurorehabilitation. The experiential neurorehabilitation therapeutic approach extends the understanding of the therapeutic process to the whole living system and its dynamics with the environment, where subjective experience plays a relevant role. It furthermore, and perhaps more significantly, opens possibilities for creating new therapeutic strategies through its theoretical precepts. The paragraphs below will provide more detail into how experiential neurorehabilitation and its premises transform the objectives of cognitive neurorehabilitation.

Prigatano (1999) categorizes cognitive neurorehabilitation with two different rehabilitation objectives. The first objective, associated with its disciplinary origins, is related to rehabilitation of cognitive functions. To do so, therapeutic strategies under this paradigm are based on recovery of cognitive deficits or on learning of skills to compensate for damaged cognitive functions. In this respect, Barbara Wilson states: “At the most fundamental level, people undergoing cognitive rehabilitation require help to remediate, reduce or alleviate their cognitive deficits” (Wilson, 2002). The theoretical precept for this objective, and the therapeutic strategies applied from it, is that cognitive learning is sufficient for recovery of the person in their family, social, and work contexts. Some examples of this objective consist of people correctly performing cancelation tasks, repeating a sequence of colors, mathematical calculations, writing letters of the alphabet, opening and closing a spastic hand, pronouncing phonemes, or performing agile and fluid flexion and extension knee movements. Over the years, cognitive neurorehabilitation has proven that specific stimulation of cognitive functions is not sufficient for biopsychosocial recovery, and that more ecological rehabilitation contexts are required (Wilson, 2002); to be sure, there is a demonstrated need for rehabilitation that moves focus away from cognitive impairment recovery and mental exercises toward aspects more related to activities of daily living (Wilson, 1997). Currently, the objectives of neurorehabilitation, as well as therapeutic strategies thereof, are based on the main premise of ecological and functional values of therapy. Thus, for example, Sohlberg and Mateer state that cognitive rehabilitation “refers to the therapeutic process of increasing or improving an individual’s capacity to process and use incoming information so as to allow increased functioning in everyday life” (1989, p. 3). The aim of this rehabilitation model is independence in walking, personal autonomy (home and social), or spoken and written communication. To achieve this, therapeutic strategies include walking through the corridors of the rehabilitation center, walking around the city, cooking a meal, washing the dishes, communicating with other people under specific clinical conditions, writing a dictation with the text provided by the therapist, and learning how to handle money.

An analysis taken from the perspective of experiential neurorehabilitation regarding the objectives and approaches of cognitive neurorehabilitation will inevitably find them insufficient for biopsychosocial recovery. Its objectives are focused on rehabilitating cognitive function and functionality, and thus abandon any consideration of the subjective attributes that accompany them. Today, we have a cognitive rehabilitation whose objective is, for example, allowing people to walk autonomously in the street, but precludes the sensation of walking, of holding your partner’s hand or picking up your grandchildren from school. It is a therapy that encourages autonomy in the kitchen, but ignores whether a person with brain damage will cook for guests or for their daughter’s school lunch. It is a therapeutic model that looks to improve written and spoken communication, but disregards whether the person has to write a letter of apology to their sister or express nostalgia when talking to their childhood friends.

Incorporating an enactive paradigm vision into neurorehabilitation changes the objectives of such therapy. Given the premises of this paradigm – where corporeality and subjectivity are essential constitutive parts of the human being – rehabilitation must maintain these precepts as fundamental objectives. For experiential neurorehabilitation, disability is an experience of biological and subjective dimensions, interdependent, which cannot be reduced or separated from each other. The essence of the rehabilitation process is to recover the concrete and singular experience of the person with disability, composed of physical action and its personal meaning. Here mobility rehabilitation is no longer just about getting the person to walk autonomously around the city; rather, it considers overcoming any feelings of fear, of falling to the ground, replacing it with sensations of walking with a spouse and children in the park, going out with friends to participate in a life-long football team membership, or strolling across the countryside in an exercise of solitude and intimacy. Here communication rehabilitation is no longer the ability to engage in conversations with others, respecting their turn to speak, and correctly explaining ideas; rather, it also approaches a recovery of the happiness one feels when recounting to one’s friends a return to work, the feeling of intimacy when reading one’s children a story at night, or the low self-esteem one may feel when unable to explain oneself as properly as one would like. Recovery from increased self-care no longer consists only of correctly sequencing the steps to make a sandwich; rather, it includes reclaiming the meaning of knowing that the sandwich is for your child to take to school, or addressing the frustration and anger you feel when you are not able to do so correctly. The rehabilitation of memory is not merely recovering a specific life event, but to again feel the emotions and meanings that accompany that experience, such as the thrill of the day your child was born, or the happiness of vacations spent with friends.

## Author Contributions

The author confirms being the sole contributor of this work and has approved it for publication.

## Conflict of Interest

The author declares that the research was conducted in the absence of any commercial or financial relationships that could be construed as a potential conflict of interest.

## Footnotes

1 In this manuscript the terms diagnosis, evaluation and treatment are used as follows. Diagnosis is the process developed by physician (e.g., neurologists and neurosurgeons) to identify neurological conditions and sequelae. Evaluation refers to instances of therapeutic examination for the purpose of supporting diagnosis, treatment planning, and treatment evaluation. The term treatment refers to therapeutic interventions that are aimed at improving, or compensating for, sequelae in those with brain damage. Both the concepts of evaluation and treatment are related to the disciplines of neuropsychology, physical therapy, occupational therapy, and speech/language therapy.

2 There are some exceptions to this rule, such as, for example, Trunk Rotation Therapy, in the neuropsychological treatment of hemispatial neglect. This syndrome is characterized by people unable to report or respond to stimuli from one side of the body (usually the left) and when such deficits cannot be attributed to a motor or sensory dysfunction but to a cognitive one. The rehabilitation of this cognitive disorder in Trunk Rotation uses body posture to rotate the trunk toward the left side, which improves stimuli integration of visual detection, brain activity, and daily activities (Spinelli and Di Russo, 1996; Wiart et al., 1997; Manly et al., 2010).

3 Today this view has been largely superseded by some models of social neuroscience and affective neuroscience. These disciplines consider the insular cortex, or fifth lobe of the brain, relevant to the perception of internal body signals and environmental stimuli (social context), which are functionally related to areas of the prefrontal and temporal cortices. An example of the importance of body states in cognition are given in Antonio Damasio and other somatic marker researchers (Damasio et al., 1991; Damasio, 1994; Damasio et al., 1996). The neural network model of social context, described by Ibáñez and Manés (2012), explains the relevance of the social environment in brain processing.

4 In contemporary neurorehabilitation literature, the term awareness or self-awareness is defined as “the capacity to perceive the ‘self’ in relatively ‘objective’ terms while maintaining a sense of subjectivity” (Prigatano and Schacter, 1991, p. 13).

5 Today in the basic sciences, especially in social and affective neuroscience, it is accepted that there are other cognitive domains that could be closer to the non-rational aspects of the subject, such as creativity, empathy, social cognition, recognition of emotions, and morality. However, in the clinical field, these cognitive domains are practically unexplored.

6 Some examples are embodied cognition, enaction, extended mind, embedded mind, or affective mind (Varela et al., 1993; Clark and Chalmers, 1998; Gallagher and Zahavi, 2008; Ward and Stapleton, 2012). Each approach has unique characteristics; some emphasize the importance of environment in cognition, while others give greater relevance to body structure (Rowlands, 2010). The current literature considers these approaches logically independent from each other, but are sometimes also combined (Ward and Stapleton, 2012).

7 The cognitive sciences present various proposals for third and first-person data joint research, such as neurophenomenology (Varela, 1996), the affective neuro-physio-phenomenology (Colombetti, 2013), and the cardiophenomenology (Depraz and Desmidt, 2018). Some of this research has been conducted in clinical contexts (e.g., Price and Aydede, 2005; Petitmengin et al., 2006; Petitmengin et al., 2007).

8 In the field of neurorehabilitation, various studies have been carried out in the phenomenological tradition (e.g., Starkstein and Lischinsky, 2002; Howes et al., 2005; O’Callaghan et al., 2006; Owen et al., 2017). There have also been different rehabilitation programs based on access to consciousness (e.g., introspection) that prioritize subjective experience (Ernst et al., 2018).

9 Francisco Varela used the expression “gaining intimacy with the domain of investigation” to refer to the second phase of phenomenological reduction (Gallagher and Brøsted Sørensen, 2006; Olivares et al., 2015).
